# Association between coffee consumption and risk of bladder cancer in a meta-analysis of 16 prospective studies

**DOI:** 10.1186/s12986-019-0390-3

**Published:** 2019-09-13

**Authors:** Zhi-Wei Dai, Ke-Dan Cai, Fu-Rong Li, Xian-Bo Wu, Guo-Chong Chen

**Affiliations:** 1 Department of Nephrology, HwaMei Hospital, University of Chinese Academy of Sciences, Ningbo, 315010 China; 2 Department of Epidemiology, School of Public Health, Southern Medical University (Guangdong Provincial Key Laboratory of Tropical Disease Research), Guangzhou, 510000 China; 3 Department of Epidemiology and Population Health, Albert Einstein College of Medicine, 1300 Morris Park Avenue, Bronx, NY 10461 USA

**Keywords:** Coffee, Bladder cancer, Cohort studies, Meta-analysis

## Abstract

**Background:**

Current evidence remains equivocal as to whether and how consumption of coffee may be associated with risk of bladder cancer, and potential influence of confounding by smoking on this association is yet to be elucidated. We conducted an updated meta-analysis of prospective studies to address these issues.

**Methods:**

Relevant studies were identified by searching PubMed and EMBASE databases from inception to April 2019. A random-effects model was used to estimate summary relative risk (RR) with corresponding 95% confidence interval (CI) of bladder cancer associated with coffee consumption.

**Results:**

The final analysis included 16 prospective studies comprising 2,122,816 participants and 11,848 bladder cancer cases. Overall, coffee consumption was not associated with risk of bladder cancer (RR _high-vs-low_ = 1.07, 95% CI: 0.96–1.20). The lack of association persisted in the strata defined by sex or participants’ smoking status. Meta-regression analyses identified the number cases (*P*_difference_ = 0.06) and the degree of adjustment for smoking (*P*_difference_ = 0.04) as potential sources of heterogeneity. There was an increased risk of bladder cancer related to higher coffee consumption among studies with fewer cases (RR _high-vs-low_ = 1.38, 95% CI: 1.05–1.81) and among those with poorer adjustment for smoking (RR _high-vs-low_ = 1.48, 95% CI: 1.14–1.93). Results were similar in the dose-response analyses (RR _1 cup/d_ = 1.01, 95% CI: 0.98–1.03).

**Conclusion:**

Best evidence available to date does not support an independent association between coffee consumption and bladder cancer risk. Some direct associations observed in individual studies may be a result of residual confounding by smoking.

**Supplementary information:**

**Supplementary information** accompanies this paper at 10.1186/s12986-019-0390-3.

## Background

Coffee is among the most commonly consumed beverages worldwide. As such, a small impact of coffee drinking on health risk could lead to significant public health consequences. Over the past decades, many epidemiologic studies have been carried out to extensively evaluate potential influences coffee drinking on multiple health outcomes, including the developments of various types of cancer [1]. While inverse associations between coffee consumption and risk of cancers at specific sites including liver and endometrium have been documented [2], some evidence for an increased risk of bladder cancer related to coffee drinking also emerged. In 1991, the International Agency for Research on Cancer (IARC) Monographs classified coffee as “possibly carcinogenic” to the bladder [3]. The IARC Monographs reviewed the accumulative evidence on coffee and cancer in 2016 and updated the statement that “overall coffee drinking was evaluated as unclassifiable as to its carcinogenicity to humans” [4]. Nevertheless, the Monographs also called for additional large prospective studies carefully accounting for important confounders, especially cigarettes smoking that is often correlated strongly with coffee drinking, for more definite conclusions.

Since the release of the 2016 statement, five large prospective studies [5–9] examining the association between coffee consumption and risk of bladder cancer have been published, comprising more than 1.45 million participants and 9000 bladder cancer cases. More importantly, four [5–8] of the five studies additionally examined the association among never-smoking participants. These data allow for an independent evaluation of coffee-bladder cancer association among never smokers to completely avoid residual confounding by smoking. Therefore, we performed a large updated meta-analysis to assess the association between coffee consumption and risk of bladder cancer.

## Methods

### Literature search

We performed a literature search in PubMed and EMBASE databases for potentially relevant studies published from inception to April 2019. We used the search term “coffee” in combination with “bladder cancer”, “urinary cancer”, or “urothelial carcinoma”, without imposing language restrictions. The detailed search strategies are reported in Additional file 1. To identify any additional studies, we also hand searched the bibliographies of retrieved full publications and those of several previous meta-analyses of coffee consumption and risk of bladder cancer [10–13]. The eligibility of the identified records was accessed independently by two authors. Any disagreement between the authors was resolved by group discussion.

### Study selection

Potentially eligible studies were prospective studies (i.e. prospective cohort, case-cohort, or nested case-control studies for which consumption of coffee were recorded prior to cancer diagnosis) that evaluated the relationship between coffee consumption and risk of incident or fatal bladder cancer. To be included in the meta-analysis, studies also had to report risk estimates such as hazard ratio, relative risks (RR), or odds ratio with corresponding 95% confidence intervals (CI) of bladder cancer associated with coffee consumption, or report relevant data for deriving these estimates. We did not exclude studies that had small sample sizes or poor adjustments for potential confounders; instead, we evaluated the impacts of these study characteristics in predefined subgroup analyses.

### Data extraction

Using a standardized data-collection form, we extracted the following characteristics from each included study: the first author’s last name, publication year, country, sources of study population, years of follow-up, number of participants, age and sex of participants, the proportion of current smokers among participants, number of bladder cancer cases, and proportion of male cases. We further extracted information on different categories of coffee consumption and the corresponding fully adjusted risk estimates with 95% CIs (by smoking status, if available), as well as adjustments for potential confounders in the statistical models. Because the association between coffee consumption and risk of bladder cancer could be particularly prone to confounding by cigarette smoking, we extracted detailed information on how smoking habits were adjusted for in the analyses (e.g. smoking status, frequency, and duration). Data were extracted by one author (Z-WD) and independently verified by other two authors (K-DC and F-RL), with any disagreement resolved by group discussions.

### Statistical analysis

Given the substantial differences in the levels of coffee consumption among individual studies, we calculated both summary risk estimates of bladder cancer for the extreme categories (i.e. highest vs. lowest) and for each 1 cup/d increment of coffee consumption. We combined study-specific risk estimates using a random-effects model to account for both within- and between-study variations [14]. For studies [15–17] in which only sex-specific rather than overall results were reported, we pooled the sex-specific estimates for each study using a fixed-effect model and included the combined results in the main analyses to maintain the correct degrees of freedom for tests of heterogeneity. For one study [16] in which the reference group was not the lowest coffee consumption, and we set the lowest category as the new reference category and calculated new risk estimates according to the method of Hamling et al [18]. For two studies [15, 19] in which some RRs of bladder cancer were reported without 95% CIs, the CIs were also derived using the Hamling et al’s methods.

If dose-response relationships between coffee consumption (e.g. 1 cup/d) and risk of bladder cancer was not examined in the original studies, we calculated the estimates using the generalized least squares trend estimation [20]. Accordingly, the levels of coffee consumption, the distributions of cases and person-years, as well as risk estimates of bladder cancer with corresponding 95% CIs for each category of coffee consumption were extracted for the estimation of a dose-response association. For a European study [21] in which coffee consumption was expressed in daily volume (ml/d) rather than cups, we rescaled the consumption into cup/d by using 125 ml as a standard size of a cup (validated in another European study [16]). When the number of cases or person-years in each coffee category was not reported, we estimated the distributions from total number of cases or person-years. The median or mean consumption in each category was used as the average amount, and when these values were not reported, the midpoint of the upper and lower boundaries was considered average amount of consumption. If the highest or lowest category was open-ended, we assumed the width of the interval to be the same as in the closest category. The results of linear dose-response analyses are presented for a 1 cup/d increment of coffee consumption. We examined potential nonlinear relationships between coffee consumption and risk of bladder cancer using restricted cubic splines, modeling three knots at percentiles 10, 50 and 95% of the distribution of coffee consumption [22]. A *P* value for nonlinearity was calculated by testing the null hypothesis that the coefficient of the second spline is equal to zero.

To explore potential sources of heterogeneity, we conducted stratified and meta-regression analyses according to the following study and population characteristics: geographic region, duration of follow-up, number of participants, sex of participants, percentage of current smoker among the study population, number of bladder cancer cases, percentage of male cases, smoking status, and adjustment for potential confounders. To better capture the different degree of being subject to confounding by smoking across individual studies, we categorized the adjustments for cigarettes smoking into three groups including: poorer adjustment for smoking: no adjustment for smoking or adjustment for smoking status only; moderate adjustment for smoking: adjustment for smoking status in addition smoking frequency (e.g. cigarettes smoked per day, or pack-years of smoking) either continuously or categorically; better adjustment for smoking: adjustment for smoking status, smoking frequency, in addition to smoking duration or lifetime smoking intensity.

Heterogeneity among studies was assessed using I^2^ statistics [23], with a value of < 25%, 25–50 and > 50% indicating little or no, moderate, and high heterogeneity, respectively. Potential publication bias was assessed using both Begg rank correlation test and Egger linear regression test [24, 25]. All statistical analyses were carried out using STATA version 12.0 (STATA Corp., College Station, TX, USA). All *P* values were two-sided, and the level of significance was at < 0.05, unless explicitly stated.

## Results

### Study selection

**Fig. 1:**
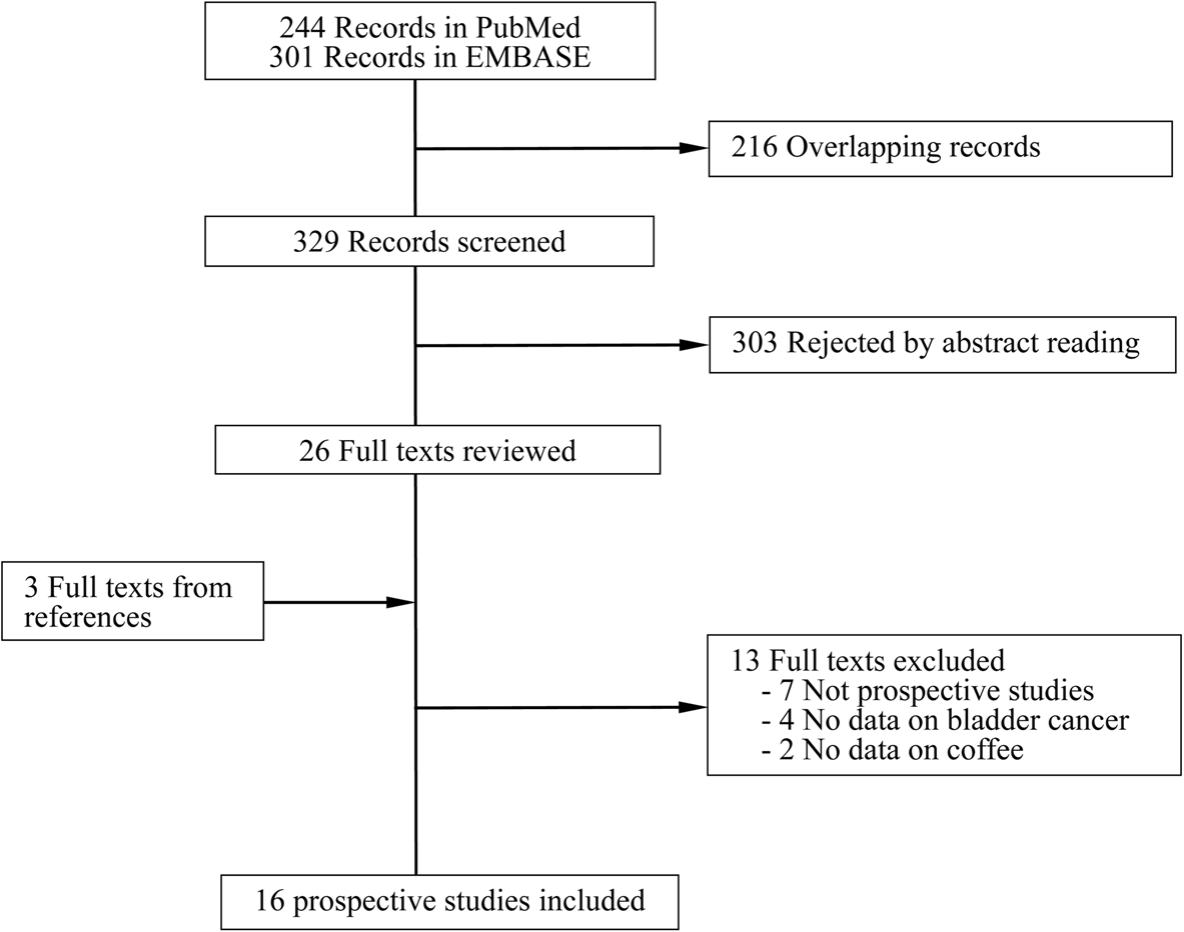
Flow chart of study selection

A flow chart of study selection is reported in Fig. 1. Briefly, a total of 329 independent reports were identified after removing overlapping records, of which 26 were retrieved for more detailed evaluations. Three additional reports were found by screening the references of relevant publications. Thirteen of the 29 reports were excluded after reviewing the full texts. As a result, 16 publications [5–9, 15–17, 19, 21, 26–31] including 16 prospective studies were included in the meta-analysis (Sugiyama et al [7] and Lukic et al [8] both combined two independent cohorts, and we counted both as one study because no cohort-specific results were presented). Overall, the 16 prospective studies included 2,122,816 participants and 11,848 bladder cancer cases.

### Study characteristics

The characteristics of the included studies are summarized in Table 1. Of the 16 studies, seven were from the US, six were from Europe, and three from Japan. Duration of follow up (5.3 to 23.4 yr), number of participants (3113 to 696,391), and number of cases (52 to 6012) ranged substantially across individual studies. Three studies included men only (one study [9] included totally male smokers), one study included women only, and the remaining studies recruited participants of both sexes. All but one study [5] used bladder cancer incidence as the study outcome. For the study [5] that focused on cancer mortality, only results for nonsmoking participants (former and/or never smokers) were reported. Thus, proportion of current smokers ranged from 0 to 100% among participants of the included studies. Reported results, statistical adjustments for potential confounders, as well as detailed information on adjustments for smoking behaviors for the included studies are summarized in Table 2. Among the 16 studies, three, eight, and five studies were deemed to have poorer, moderate, and better adjustments for cigarettes smoking, respectively.

**Table 1:** Characteristics of prospective studies included in the meta-analysis

Author, years	Country	Population	Duration, *yr*	Participants			N of cases (% M)
				N and sex	Age, *yr*	% Smoker	
Jacobsen, 1986 [19]	Norway	Norwegian cohort of mostly men	11.5	13,664 M; 2891 W	≥35	37.9% (M only)	94 (69.1%)
Mills, 1991 [26]	US	AHS	5.3	34,198 M&W	≥25	1.4%	52 (69.2%)
Chyou, 1993 [27]	US	Japanese-American men	22.0	7995 M	NR	43.7%	96 (100%)
Stensvold, 1994 [15]	Norway	CVD screening Participants	10.1	21,735 M; 21,238 W	35–54	46.1% (M); 34.1% (W)	53 (75.5%)
Michaud, 1999 [28]	US	HPFS	9.1	47,909 M	40–75	9.6%	252 (100%)
Nagano, 2000 [29]	Japan	LSS	11.7	14,873 M; 23,667 W	52.8 (M); 56.8 (W)	35.0%	114 (72.8%)
Zeegers, 2001 [16]	The Netherlands	NCS	6.1	1515 M; 1598 W (sub-cohort)	55–69	34.0% (M); 20.0% (W)	569 (93.5%)
Tripathi, 2002 [30]	US	IWHS	13.0	37,459 W	55–69	15.0%	112 (0%)
Kurahashi, 2009 [17]	Japan	JPHC	12.6	49,566 M; 54,874 W	40–69	52.5% (M); 6.7% (W)	206 (79.6%)
Ros, 2011 [21]	5 European countries	EPIC	9.3	67,914 M; 165,322 W	53.7 (M); 52.9 (W)	23.7%	513 (50.5%)
Hashibe, 2015 [31]	US	PLCO	14.0	97,334 M&W	55–74	9.1%	398 (NA)
Loftfield, 2017 [6]	US	NIH-AARP Diet and Health	15.5	469,047 M&W	50–71	14.2%	6012 (84.6%)
Sugiyama, 2017 [17]	Japan	MCS; OCS	17.6 (MCS); 13.3 (OCS)	73,346 M&W	40–64 (MCS); 40–79 (OCS)	35.3%	274 (73.7%)
Gapstur, 2017 [5]	US	CPS-II	23.4	696,391 M&W nonsmokers	28–94	0%	1789 BCa death (NA)
Lukic, 2018 [8]	Norway and Sweden	NOWAC; NSHDS	13.6	193,439 M&W	25–74	24.8%	479 (59.7%)
Hashemian, 2019 [9]	Finland	ATBC	17.6	26,841 male smokers	57.2	100%	835 (100%)

Abbreviations: *AHS* Adventist Health Study, *ATBC* Alpha-Tocopherol, Beta-Carotene Cancer Prevention, *BCa* bladder cancer, *CPS* Cancer Prevention Study, *CVD* cardiovascular disease, *EPIC* European Prospective Investigation into Cancer and Nutrition Study, *HPFS* Health Professionals Follow-up Study, *IWHS* Iowa Women’s Health Study, *JPHC* Japan Public Health Center, *LSS* Life-Span Study, *M* men, *MCS* Miyagi Cohort Study, *NA* not available, *NCS* Netherlands Cohort Study, *NIH-AARP* National Institutes of Health-American Association of Retired Persons, *NOWAC* Norwegian Women and Cancer, *NSHDS* Northern Sweden Health and Disease Study, *OCS* Ohsaki Cohort Study, *PLCO* Prostate, Lung, Colorectal, and Ovarian, *W* women

**Table 2:** Reported results and statistical adjustments in the prospective studies included in the meta-analysis

Author, years	Consumption, *highest* vs. *lowest*	RR (95% CI), *highest* vs. *lowest*	Covariate adjustment	Smoking adjustment
Jacobsen, 1986 [19]	≥7 vs. ≤ 2 cups/d	0.99 (0.53–1.86) 0.98 (0.47–2.03)(M)	Age, sex, residence, and smoking (for M only)	Never, former, current (1–9, 10–19, ≥20 cig/d) (for M only)
Mills, 1991 [26]	≥2 cups/d vs. never	1.99 (0.91–4.34) 2.03 (0.70–5.87)(NS) 1.14 (0.46–2.80)(FS/CS)	Age, sex, and smoking	Never, former, current
Chyou, 1993 [27]	≥5 vs. ≤ 1 times/wk	2.07 (0.84–5.12)	Age and smoking	Pack-years (0, > 0–30, > 30)
Stensvold, 1994 [15]	≥7 vs. ≤ 2 cups/d	1.50 (0.45–5.02)(M) 2.40 (0.28–20.5)(W)	Age, residence, and smoking	Cig/d (continuous)
Michaud, 1999 [28]	≥4 cups/d vs. < 1 cup/mo	0.79 (0.48–1.30)	Age, region, energy intake, fruit and vegetable intake, and smoking	Smoking status (smoker, nonsmoker) and pack-years (6 categories)
Nagano, 2000 [29]	≥5 vs. 0 times/wk	0.90 (0.52–1.56)	Age, sex, radiation dose, education, BMI, calendar time, and smoking	Never, former, current (≤20, > 20 cig/d)
Zeegers, 2001 [16]	≥7 (M)/≥5(W) vs. < 2 cups/d	1.36 (0.82–2.04)(M) 0.32 (0.15–0.68)(W)	Age, tea consumption, and smoking	Cig/d (continuous), years of smoking (continuous)
Tripathi, 2002 [30]	≥4 cups/d vs. < 1 cup/mo	1.59 (0.95–2.68)	Age	None
Kurahashi, 2019 [17]	≥3(M)/≥1(W) cup/d vs. almost never	1.37 (0.75–2.51)(M) 0.55 (0.23–1.33)(W) 2.48 (0.88–7.05)(NS)(M) 2.09 (0.96–4.54)(FS)(M) 2.24 (1.21–4.16)(NS/FS)(M) 1.13 (0.65–1.97)(CS)(M)	Age, area, alcohol, green tea consumption, and smoking	Never, former, current (< 25, ≥25 pack-years)
Ros, 2011 [21]	≥875(M)/500(W) ml/d vs. < 429 (M)/250(W) ml/d	1.11 (0.85–1.43)	Age, sex, center, energy intake, and smoking.	Smoking status (never, former and current), duration (continuous), and lifetime intensity (continuous)
Hashibe, 2015 [31]	≥2 vs. < 1 cup/d	1.08 (0.85–1.39)	Age, sex, race, education, and smoking	Smoking status (never, former, current), frequency (1–10, 11–20, 21–30, > 30 cig/d), duration (1–10, 11–20, > 20 yr), years since quitting (> 0–2, 3–5, 6–10, 11–20, > 20 yr).
Loftfield, 2017 [6]	≥4 cup/d vs. none	1.18 (1.05–1.33) 1.25 (1.09–1.43)(M) 0.97 (0.74–1.25)(W) 0.87 (0.65–1.17)(NS) 1.23 (1.04–1.33)(FS) 1.32 (0.95–1.81)(CS)	Age, sex, race/ethnicity, BMI, education, reported health status, fruit intake, vegetable intake, supplement use, physical activity, diabetes, family history of cancer, and smoking	Pipes or cigars (ever, never), smoking frequency (1–10, 11–20, 21–30, 31–40, 41–60, ≥60 cig/d), years since quitting (≥1–4, 5–9, ≥10 yr).
Sugiyama, 2017 [7]	≥3 cup/d vs. none	0.56 (0.32–0.99) 0.57 (0.31–1.07)(M) 0.44 (0.10–1.97)(W) 0.62 (0.14–2.72)(NS) 0.61 (0.32–1.17)(FS/CS)	Age, sex, BMI, hypertension, diabetes, MI, stroke, job status, education, alcohol, green tea consumption, walking, and smoking	Never, former, current (< 20, ≥20 cig/d)
Gapstur, 2017 [5]	≥6 cup/d vs. never	0.89 (0.73–1.09)(NS/FS) 0.80 (0.57–1.12)(NS) 0.97 (0.74–1.27)(FS)	Age, sex, race, marital status, education, alcohol consumption, BMI, physical activity, family history of cancer, red and processed meat intake, vegetable intake, tea consumption, and smoking.	Years since quitting (< 10, 10- < 20, ≥20 yr) and cig/d (< 20, 20–29, ≥30).
Lukic, 2018 [8]	≥4 vs. < 1 cup/d	1.34 (0.94–1.90) 1.23 (0.78–1.95)(M) 1.46 (0.84–2.51)(W) 1.87 (1.01–3.45)(NS) 1.18 (0.77–1.81)(FS/CS)	Age, sex, and smoking	Never, former, current
Hashemian, 2019 [9]	≥4 vs. < 1 cup/d	1.10 (0.81–1.49)(CS)	Age, education, alcohol, diabetes, physical activity, fruit intake, vegetable intake, tea consumption, and smoking	Smoking years (continuous), cig/d (continuous)

Abbreviations: *BMI* body mass index, *cig* cigarettes, *CS* current smoker, *FS* former smoker, *M* men, *MI* myocardial infarction, *mo* month, *NS* never smoker, *W* women, *wk*, week

^a^ 95% CIs were calculated using raw data

^b^ Data were rescaled by using the lowest consumption group as the reference

### Meta-analysis

**Fig. 2:**
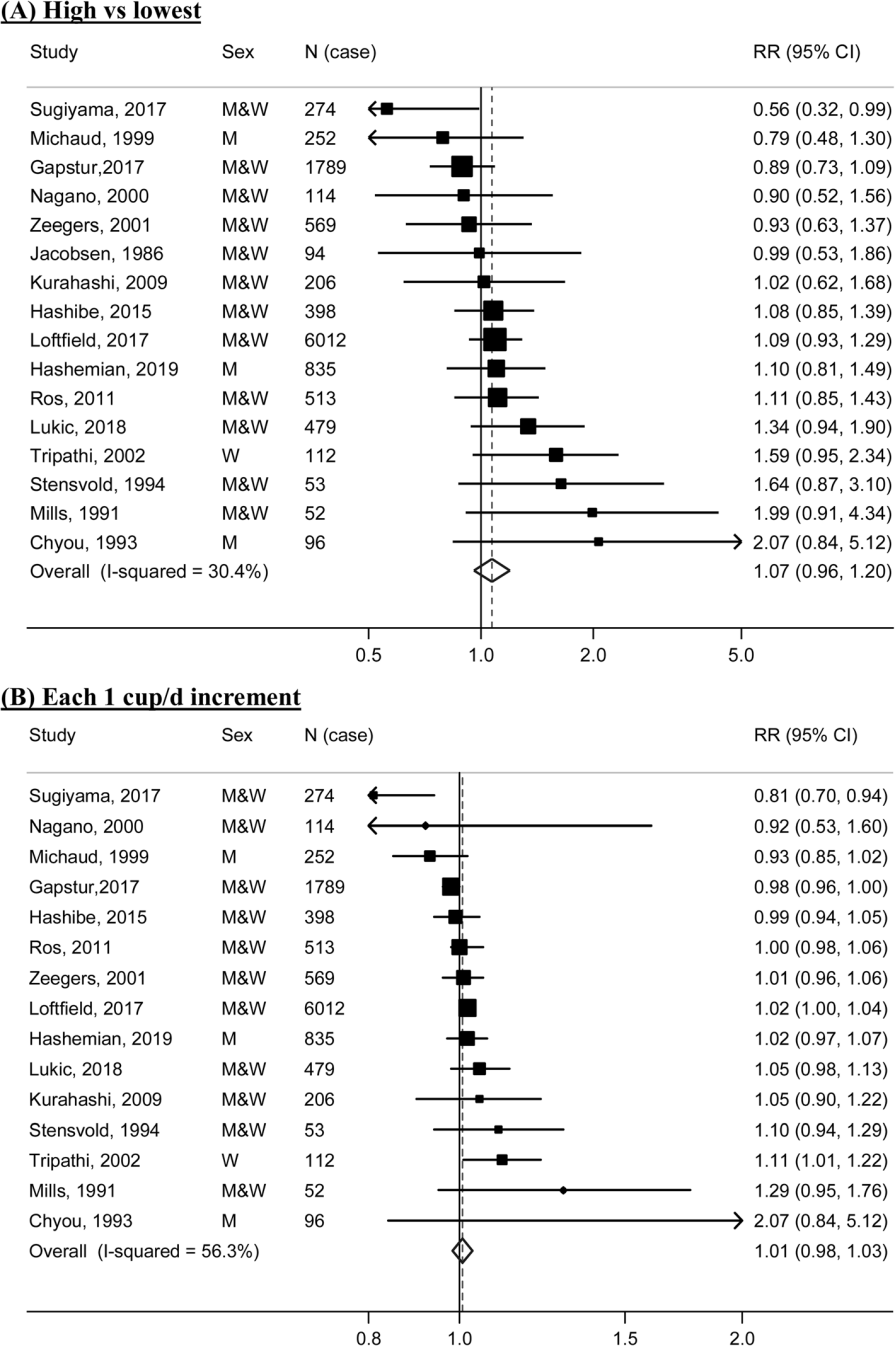
Forest plots for the meta-analysis of coffee consumption and risk of bladder cancer. **a** highest vs. lowest analysis; **b** dose-response analysis of 1 cup/d increment. M, men; W, women

A meta-analysis of the 16 prospective studies yielded a summary RR of 1.07 (95% CI: 0.96–1.20) for the highest compared with the lowest categories of coffee consumption (Fig. 2a). One study [19] did not provide eligible data for the dose-response analysis. A dose-response meta-analysis of the remaining 15 studies showed a summary RR of 1.01 (95% CI: 0.98–1.03) for each 1 cup/d increment of coffee consumption (Fig. 2b). Moderate heterogeneity was observed in both high-vs-low (*I*^2^ = 30.4%) and dose-response analyses (*I*^2^ = 56.3%). There was no evidence of a nonlinear association between coffee consumption and risk of bladder cancer (*P*_nonlinearity_ = 0.65).

### Subgroup and sensitivity analyses

Results of subgroup analyses according various predefined study and population characteristics are shown in Table 3. For most of these subgroup populations, coffee consumption was not significantly associated with risk of bladder cancer, either in the high-vs-low or in the dose-response analyses. Number of bladder cancer cases (*P*_difference_ = 0.06 and 0.04 in the high-vs-low and dose-response analyses, respectively) in addition to the degree of adjustment for smoking (*P*_difference_ = 0.04 and 0.06 in the high-vs-low and dose-response analyses, respectively) were suggested as potential sources of heterogeneity. A stronger positive association between coffee consumption and risk of bladder cancer was observed for studies with smaller (< 200) number of cases (RR _high-vs-low_ = 1.38, 95% CI: 1.05–1.81; RR _1 cup/d_ = 1.12, 95% CI: 1.04–1.21), and for those with poorer adjustment for smoking (i.e. without adjustment, or with adjustment for smoking status only) (RR _high-vs-low_ = 1.48, 95% CI: 1.14–1.93; RR _1 cup/d_ = 1.08, 95% CI: 1.02–1.15). Adjustment for tea consumption also emerged as a potential source heterogeneity in the high-vs-low analysis (*P*_difference_ = 0.042) but not in the dose-response analysis (*P*_difference_ = 0.26).

**Table 3:** Subgroup and sensitivity analyses

	Highest vs. lowest consumption				Each 1 cup/d increment			
	*N*	RR (95% CI)	*I*^2^ (%)	*P*-diff	*N*	RR (95% CI)	*I*^2^ (%)	*P*-diff
*Subgroup analyses*								
Geographic region								
US	7	1.10 (0.92–1.32)	50.4	*Ref.*	7	1.01 (0.97–1.04)	70.0	*Ref.*
Europe	6	1.13 (0.98–1.31)	0	0.60	5	1.02 (0.99–1.04)	0	0.55
Japan	3	0.81 (0.57–1.16)	23.1	0.23	3	0.92 (0.75–1.14)	65.3	0.30
Duration of follow-up								
≥ 10 yr	12	1.08 (0.95–1.23)	35.9		11	1.01 (0.98–1.04)	62.6	
< 10 yr	4	1.04 (0.81–1.35)	31.4	0.86	4	1.00 (0.95–1.04)	42.3	0.67
No. of participants								
≥ 50,000	8	1.02 (0.91–1.15)	28.9		8	1.00 (0.98–1.03)	61.1	
< 50,000	8	1.21 (0.96–1.51)	28.9	0.26	7	1.04 (0.97–1.13)	52.0	0.67
Sex								
Men	10	1.16 (1.00–1.35)	21.5		9	1.01 (0.98–1.05)	42.0	
Women	7	0.90 (0.59–1.37)	63.6	0.37	6	0.98 (0.89–1.08)	56.0	0.76
No. of cases								
≥ 200	10	1.02 (0.92–1.13)	19.0		10	1.00 (0.98–1.02)	57.3	
< 200	6	1.38 (1.05–1.81)	12.0	0.064	5	1.12 (1.04–1.21)	0	0.037
% Male cases								
≥ 75%	6	1.10 (0.95–1.26)	4.1		6	1.02 (0.94–1.10)	69.3	
< 75%	9	1.10 (0.92–1.30)	37.6	0.98	7	1.01 (0.99–1.04)	18.5	0.87
% Current smoker								
≥ 25%	8	1.02 (0.82–1.25)	26.3		7	1.00 (0.94–1.07)	52.6	
< 25%	8	1.10 (0.96–1.26)	40.5	0.60	8	1.01 (0.98–1.03)	63.6	0.98
Smoking status								
Never	6	1.15 (0.79–1.67)	56.5	*Ref.*	6	1.02 (0.95–1.09)	58.0	*Ref.*
Former	3	1.18 (0.92–1.52)	55.5	0.80	3	1.01 (0.97–1.05)	69.7	0.99
Never/former	6	1.20 (0.93–1.56)	61.7	0.71	6	1.03 (0.98–1.08)	76.3	0.80
Current	3	1.19 (0.97–1.46)	0	0.72	3	1.04 (1.01–1.07)	0	0.50
Former/current	4	1.12 (0.88–1.43)	33.3	0.81	4	1.01 (0.95–1.07)	38.4	0.73
Statistical adjustment								
Smoking^a^								
Poorer	3	1.48 (1.14–1.93)	0	*Ref.*	3	1.08 (1.02–1.15)	8.9	*Ref.*
Moderate	8	1.00 (0.80–1.26)	31.7	0.058	7	0.98 (0.90–1.07)	60.5	0.17
Better	5	1.03 (0.93–1.13)	0	0.042	5	1.00 (0.98–1.02)	49.7	0.055
Alcohol drinking								
No	12	1.13 (1.01–1.26)	10.4		11	1.02 (0.99–1.04)	34.5	
Yes	4	0.91 (0.74–1.13)	34.2	0.067	4	0.98 (0.92–1.04)	68.8	0.075
Education								
No	10	1.18 (1.00–1.40)	19.6		9	1.03 (0.99–1.07)	44.1	
Yes	6	0.99 (0.87–1.13)	31.5	0.14	6	0.99 (0.96–1.03)	69.5	0.33
Physical activity								
No	13	1.10 (0.95–1.29)	33.4		12	1.01 (0.97–1.05)	53.6	
Yes	3	1.02 (0.88–1.17)	24.4	0.48	3	1.00 (0.97–1.04)	75.7	0.90
BMI								
No	12	1.15 (1.02–1.29)	5.1		12	1.02 (0.99–1.05)	33.9	
Yes	4	0.92 (0.74–1.14)	52.9	0.099	3	0.98 (0.93–1.03)	80.7	0.31
Diabetes								
No	13	1.10 (0.96–1.25)	27.1		12	1.01 (0.98–1.04)	43.2	
Yes	3	0.97 (0.73–1.29)	60.4	0.57	3	0.99 (0.92–1.05)	78.4	0.78
Family history of cancer								
No	14	1.10 (0.96–1.27)	27.9		13	1.01 (0.98–1.05)	49.9	
Yes	2	0.99 (0.82–1.21)	57.6	0.41	2	1.00 (0.96–1.04)	87.0	0.74
Energy intake								
No	14	1.08 (0.96–1.23)	35.3		13	1.01 (0.99–1.04)	59.0	
Yes	2	1.00 (0.74–1.36)	28.9	0.74	2	0.98 (0.91–1.04)	51.3	0.43
Fruit/vegetable consumption								
No	12	1.13 (0.97–1.33)	32.1		11	1.02 (0.98–1.06)	51.5	
Yes	4	1.00 (0.88–1.14)	16.5	0.29	4	1.00 (0.97–1.03)	72.1	0.54
Tea consumption								
No	11	1.15 (1.02–1.29)	11.4		10	1.02 (0.99–1.05)	40.8	
Yes	5	0.92 (0.96–1.20)	12.3	0.042	5	0.99 (0.96–1.03)	62.1	0.26
*Sensitivity analysis*								
Excluding 1 study^b^	15	1.10 (0.98–1.23)	22.4		14	1.01 (0.99–1.04)	47.0	
Excluding 2 studies^c^	14	1.10 (0.97–1.25)	27.9		13	1.01 (0.98–1.04)	51.0	

“*N*” indicates the number of studies included in the analyses; “*P*-diff” indicates *P* values for differences between subgroup population (derived using meta-regression analyses)

^a^ Poorer adjustment for smoking: no adjustment for smoking or adjustment for smoking status only; moderate adjustment for smoking: adjustment for smoking status in addition smoking frequency (e.g. cigarettes smoked per day, or pack-years of smoking) either continuously or categorically; better adjustment for smoking: adjustment for smoking status, smoking frequency, in addition to smoking duration or lifetime smoking intensity. One study (Gaspstur, 2017) that reported results only for nonsmokers (never and former smokers) and adjusted for smoking history for former smokers was included in the “better” group. Another study (Hashemian, 2019) that included totally current smokers and adjusted for both smoking frequency and duration was included in the “moderate group” (this study further examined coffee-bladder cancer association by smoking frequency and did not find group differences in the association)

^b^ Excluding one study (Gaspstur, 2017) in which the study outcome was bladder cancer mortality, and all analyzed participants were never or former smokers

^c^ In addition to the above-mentioned study (Gaspstur, 2017), further excluding another study (Hashemian, 2019) in which all participants were current smokers

Combined results from six prospective studies [5–8, 17, 26] suggested that coffee consumption was not associated with bladder cancer risk among never smokers (RR _high-vs-low_ = 1.15, 95% CI: 0.79–1.67; RR _1 cup/d_ = 1.02, 95% CI: 0.95–1.09). The lack of association persisted in other smoking categories, although dose-response (but not high-vs-low) analysis suggested modestly increased risk among current smokers. Results of the meta-analysis were similar after excluding one study [5] looking at cancer mortality and another study [9] fully consisting of male smokers (Table 3).

### Publication bias

For both high-vs-low and dose-response analyses, there was no evidence of publication bias according to results of Begg test or Egger test (all *P* values > 0.40).

## Discussion

In this meta-analysis of 16 prospective studies including over 2.1 million participants and 11,000 bladder cancer cases, coffee consumption was not associated with risk of bladder. The lack of association persisted in the strata defined by various study characteristics including sex and participants’ smoking status. Meta-regression analyses identified the number cases and the degree of adjustment for smoking as potential sources of heterogeneity. Coffee consumption was associated with increased risk of bladder cancer among studies with fewer cancer cases and among those with poorer adjustment for smoking, indicating that some direct associations observed in individual studies may be a results of residual confounding by smoking.

For studies of coffee consumption and smoking-related cancers including bladder cancer, potential confounding by smoking merits particular attention. Observationally [5, 6] and genetically [32], heavier coffee consumers are more likely to be smokers. In a large cohort of US population [6], only 5.3% of coffee non-drinkers were current smokers, whereas the corresponding data in those consuming ≥4 cups/d of coffee was 25.8%. For current smokers, coffee consumption tends to be associated with larger smoking amount [6] and longer smoking duration [31]; for former smokers, habitual coffee drinkers are likely to have been quitting smoking for shorter duration [6]. All these coffee-related smoking features are associated with increased risk of bladder cancer when comparing with never smoking [33]. Thus, adjustment for smoking among individual studies needs to be carefully evaluated in meta-analyses of coffee consumption and smoking-related cancer.

Several previous meta-analysis [10–13] of published case-control and cohort studies suggested increased risk of bladder cancer associated with higher consumption of coffee. However, the overall positive association was totally driven by case-control data, and all of these meta-analyses showed no significant coffee-bladder cancer association in the cohort-only analyses (including nine [13] or fewer cohorts [10–12]), which is concordant with findings of the current meta-analysis. Of note, in the 2015 meta-analysis by Wu et al [12], it was suggested that coffee consumption was substantially associated with a 72% increased risk of bladder cancer among nonsmokers (never and former smokers). More recently, results from a pooled analysis of 13 case-control studies also suggested a positive association between coffee consumption and risk of bladder cancer in never smokers [34]. Such positive associations in these previous analyses, however, may have been largely or fully driven by residual confounding by smoking history (former smokers relative to never smokers still have increased bladder cancer risk [33]) or by serious information biases (e.g. recall bias) inherent in the original case-control studies. Being aware of earlier information that coffee may be carcinogenic for the bladder (e.g. IARC classified coffee as “possibly carcinogenic” to the bladder in 1991 [3]), patients with bladder cancer in the case-control studies may exaggerate the amount of coffee consumed. Evaluating the association among never smoking individuals using prospective data can avoid these biases. In the current meta-analysis, a subgroup analysis pooling data from six prospective studies, four of which were published after the Wu et al’ meta-analysis [12], did not support an association between coffee consumption and risk of bladder cancer among never smokers.

Among the complex mixture of hundreds of chemicals contained in coffee, caffeine has been proposed as having anti-cancer properties [35], which raises the question as to whether any inverse association between coffee consumption and bladder cancer was diluted by consumption of decaffeinated coffee. However, consumption of decaffeinated coffee was uncommon during the initiations of the most original studies, such that only a few studies were able to compare two types of coffee for the association with bladder cancer, observing no meaningful differences in the associations [5, 28]. Also, the amount of other potentially anti-carcinogenic compounds in coffee such as the coffee diterpenes cafestol and kahweol [36] my differ by the brewing methods of coffee, with higher amount in unfiltered (including boiled) coffee than in filtered or instant coffee [37]. Two prospective studies [8, 9] evaluated both filtered and boiled coffee in relation to bladder cancer risk, and neither find apparent difference in the association according to coffee brewing methods, though additional studies are still needed.

Our meta-analysis has several strengths. The prospective design of original studies eliminated the influence of recall and selection biases on the examined association. The large number of cases enhanced the statistical power and enabled us to detect any modest association between coffee consumption and bladder cancer risk. The robustness of our findings was further supported by the results of various analyses defined according to smoking status and the degree of adjustment for smoking and by the concordance of results from high-vs-low and dose-response meta-analyses. There are also some limitations to this meta-analysis. All primary studies collected information on coffee consumption only once at baseline without updating data, which may have led to regression dilution bias and thus an attenuated association. In addition, whether the association between coffee and bladder cancer may differ by roasting degree of coffee beans or brewing methods for coffee preparation [8, 9], or by histological subtypes of bladder cancer [21] cannot be evaluated in the current meta-analysis due to few or no eligible studies available. Finally, the differences in the association of coffee consumption with risk of bladder according to the number of cases or smoking adjustment were only marginally significant. Given the multiple subgroup analyses performed, the possibility of chance findings cannot be excluded and future large prospective studies with careful consideration of potential confounding by smoking are still warranted.

## Conclusion

In summary, findings from this large meta-analysis of prospective studies suggest that coffee consumption was not significantly associated with long-term risk of bladder cancer. Such a null association was similar for men and women, and was confirmed in never smokers. Thus, best evidence available to date does not support the notion that consumption of coffee may increase the risk of bladder cancer.

## Supplementary information

**Additional file 1.** Literature search strategies in the databases. (DOCX 11 kb)

## Data Availability

The datasets used and/or analyzed during the current study are available from the manuscript and the corresponding author on reasonable request.

## Abbreviations

CI: Confidence interval
IARC: the International Agency for Research on Cancer
RR: Relative risk
