# Supplementary material for: Association between coffee consumption and risk of bladder cancer in a meta-analysis of 16 prospective studies
Source: Nutr Metab (Lond). 2019 Sep 13;16:66. doi: 10.1186/s12986-019-0390-3 (PMC6743122; doi:10.1186/s12986-019-0390-3)
Supplement: Supplementary file 1 — Additional file 1. Literature search strategies in the databases. (DOCX 11 kb) [file 12986_2019_390_MOESM1_ESM.docx]

**PubMed search**

("coffee"[MeSH Terms] OR "coffee"[All Fields]) AND (("urinary bladder"[MeSH Terms] OR ("urinary"[All Fields] AND "bladder"[All Fields]) OR "urinary bladder"[All Fields] OR "bladder"[All Fields]) OR ("urinary tract"[MeSH Terms] OR ("urinary"[All Fields] AND "tract"[All Fields]) OR "urinary tract"[All Fields] OR "urinary"[All Fields]) OR urothelial[All Fields]) AND (("neoplasms"[MeSH Terms] OR "neoplasms"[All Fields] OR "cancer"[All Fields]) OR ("carcinoma"[MeSH Terms] OR "carcinoma"[All Fields]))

**EMBASE search**

('coffee'/exp OR coffee) AND ('bladder cancer'/exp OR 'bladder cancer' OR (('bladder'/exp OR bladder) AND ('cancer'/exp OR cancer)) OR 'urinary cancer' OR (urinary AND ('cancer'/exp OR cancer)) OR 'urothelial carcinoma'/exp OR 'urothelial carcinoma' OR (urothelial AND ('carcinoma'/exp OR carcinoma)))
